# FOXA1, induced by RC48, regulates HER2 transcription to enhance the tumorigenic capacity of lung cancer through PI3K/AKT pathway

**DOI:** 10.7150/jca.100210

**Published:** 2024-09-16

**Authors:** Mengyang Zhao, Ning Zhang, Yijun Wang, Kang Han, Tianhui Gao, Xue Li

**Affiliations:** 1 Department of Oncology, Henan Provincial People's Hospital, People's Hospital of Zhengzhou University, Zhengzhou, Henan, 450003, China.; 2 Department of Radiology, Henan Provincial People's Hospital, People's Hospital of Zhengzhou University, Zhengzhou, Henan, 450003, China.; 3 Ophthalmology, Henan Provincial People's Hospital, People's Hospital of Zhengzhou University, Zhengzhou, Henan, 450003, China.; 4 Department of Pathology, Henan Provincial People's Hospital, People's Hospital of Zhengzhou University, Zhengzhou, Henan, 450003, China.

**Keywords:** FOXA1, RC48, HER2, proliferation, apoptosis.

## Abstract

Lung cancer remains the tumor with the highest global incidence and mortality rates. Current primary treatment modalities encompass targeted therapy, immunotherapy, and chemotherapy; however, a subset of patients derives no benefit from these interventions. Recently, the HER2-targeting antibody drug Disitamab vedotin (RC48) was approved and introduced primarily for gastric and bladder cancers, with minimal investigation in the field of lung cancer. This study demonstrates that FOXA1 directly binds to the promoter region of HER2, influencing the HER2/PI3K/AKT signaling pathway, which consequently modulates factors that foster lung cancer proliferation and impede apoptosis. Unlike FOXA1, HER2 does not influence the expression of FOXA1. Intriguingly, in lung cancer cells, RC48 not only impacts the HER2/PI3K/AKT pathway but also affects the FOXA1/HER2/PI3K/AKT pathway, thereby exerting a robust antitumor effect. In clinical specimens, heightened expressions of FOXA1 and HER2 correlate positively with clinical progression and poorer prognosis. These findings suggest that FOXA1 may serve as a potential biomarker or therapeutic target in future non-small cell lung cancer (NSCLC) treatments, and ongoing research may position RC48 as a transformative agent in lung cancer therapy.

## Introduction

Lung cancer is the malignancy with the highest global incidence, mortality, and growth rate, posing a significant threat to human health and life^1^,^2^. At initial diagnosis, approximately one-third of NSCLC patients cannot receive radical surgical treatment due to metastasis. The primary methods of treatment include chemotherapy, targeted therapy, and immunotherapy. HER2, belonging to the epidermal growth factor receptor (EGFR) family, has no identified ligands for direct interaction. It primarily activates the MEK-ERK-MAPK pathway and the PI3K-AKT bypass by forming heterodimers with other family members for signal transmission. HER2 variations encompass gene mutations, gene amplification, and protein overexpression. Unlike the prevalent gene amplification and protein overexpression in breast and gastric cancers, NSCLC primarily exhibits HER2 variations through gene mutations^3^,^4^. The mutation rate of HER2 in NSCLC ranges between 1% and 4%, predominantly occurring in non-smokers, females, and lung adenocarcinoma patients^5^,^6^, with over 90% presenting as exon 20 insertion mutations, notably Y772_A775YVMA^7^. Currently, the first-line treatment for HER2-positive NSCLC in China typically involves platinum-based chemotherapy with anti-angiogenesis treatment, with limited approved targeted treatment medications. HER2-targeted therapeutic drugs include TKIs, anti-HER2 monoclonal antibodies, and antibody-drug conjugates (ADCs). Given the limited effectiveness of anti-HER2 monoclonal antibodies, TKIs and ADCs are anticipated to be significant treatment options for HER2-positive NSCLC.

Antibody-drug conjugates (ADCs) consist of three components: monoclonal antibodies, cytotoxic agents, and linkers. The monoclonal antibodies target and bind to specific tumor cell antigens, forming an ADC-antigen complex. This complex is internalized and degraded by lysosomes within the tumor cells, releasing the cytotoxic drug which then inhibits tumor cell proliferation. T-DM1 (trastuzumab-emtansine), a notable anti-HER2 ADC, has been used for treating breast cancer. However, its initial trial on relapsed HER2-mutated non-small cell lung cancer (NSCLC) was unsuccessful and terminated early due to limited efficacy^8^-^10^. Despite these results, T-DM1 exhibited potential in small-sample trials for advanced HER2-mutant NSCLC, indicating that enhancements are needed for the duration of its effectiveness. With advancements in ADC technology, the newer generation ADC, T-DXd (DS-8201), which targets HER2, has shown prominence due to its innovative structural design and effective anti-tumor mechanism.^11^. Given the significant improvement in survival rates with second-line treatment using ADC drugs, targeting HER2-mutant NSCLC presents a promising future direction. Consequently, a novel humanized IgG1 monoclonal antibody, Hertuzumab (RC48), which incorporates the Val-Cit dipeptide linker, was selected for evaluation. This anti-HER2 ADC, produced in China, specifically binds to the HER2 extracellular domain (ECD) and is conjugated to MMAE via cysteine residues exposed by antibody inter-chain disulfide bond reduction. While RC48 has shown efficacy in gastric, urothelial, ovarian, and breast cancers^12^-^15^, its application in lung cancer remains unreported. This study investigates RC48 as a targeted NSCLC treatment option, demonstrating enhanced anti-tumor efficacy and minimized toxicity in preliminary cynomolgus trials, positioning it as a superior choice among current therapeutic agents, including other ADCs.

Members of the FOXA family, including FOXA1, FOXA2, and FOXA3, are widely distributed across animals and fungi. This family significantly influences cell proliferation and differentiation, the development and progression of malignant tumors, and embryonic growth. Recent studies have increasingly focused on the relationship between FOXA family members and malignant tumors^16^,^17^. Different FOXA members exhibit varied effects on tumorigenesis across various cancers. They suppress tumor growth in gastric adenocarcinoma, breast cancer, liver cancer, thyroid cancer, and nasopharyngeal cancer, yet they enhance growth, invasion, and metastasis in prostate cancer^18^,^19^. Previous research has established FOXA1's critical role in lung cancer progression, particularly in non-small cell lung cancer (NSCLC), where it enhances proliferation, invasion, migration, and diminishes chemosensitivity^20^-^25^. Additionally, FOXA1 mediates the dynamic transcriptional responses to HER2-targeted therapy and binds to the HER2 gene promoter to affect transcription in breast cancer^26^,^27^. However, studies on the interaction between FOXA1 and HER2, and their impact on NSCLC patient prognosis, are scarce. This study mainly explores the relationship between FOXA1 and HER2 in lung cancer and the regulatory effects of RC48 on them, providing a potential basis for the treatment of lung cancer.

## Materials and methods

### Patients

The clinical data for 87 patients with advanced NSCLC, treated at our hospital from March 2018 to September 2019, were retrospectively analyzed. Inclusion criteria included: (1) ages 18 to 70 years; (2) pathological or cytological confirmation of advanced NSCLC; (3) no prior chemoradiotherapy; (4) availability of tumor tissue sections for pre-treatment immunohistochemical analysis of FOXA1 and HER2; and (5) complete clinical and follow-up data. Exclusion criteria were: (1) concurrent malignant tumors; (2) severe infection, inflammation, or serious complications; and (3) non-adherence to specified treatment protocols or death. Clinicopathological characteristics were derived from medical records and pathology reports (refer to Table 1). The follow-up cut-off date was 30 September 2019. This study received approval from the Medical Ethics Committee of Zhengzhou University. Each patient provided written informed consent prior to undergoing surgery.

### Immunohistochemistry

Tumor tissues were obtained from patients for pathological or cytological confirmation, sequentially sectioned at 4-μm thickness following standard fixation and embedding in paraffin. Subsequent to slide preparation, the sections underwent processing as stipulated by the protocol of the immunohistochemical detection kit. Initially, the slides were deparaffinized in xylene and subjected to a gradient of ethanol and xylene. This was followed by triple washing in phosphate-buffered saline (PBS). Peroxidase blocking solution (reagent A) was then administered, and the sections incubated at room temperature for 10 minutes before another series of three PBS washes. Thereafter, serum (reagent B) was applied and subsequently removed, followed by incubation at room temperature for one hour. Approximately 50 μL of primary antibody was dispensed onto each section, which was then incubated at 4°C overnight and subsequently triple-washed in PBS. A biotin-labeled secondary antibody (reagent C) was then added, and the sections were again incubated at room temperature for 10 minutes before a further three PBS washes. Finally, 50 μL of streptavidin-peroxidase solution (reagent D) was added; following incubation at room temperature for 10 minutes and another triple rinse in PBS, the sections were stained with DAB solution and counterstained with hematoxylin. The slides were then evaluated after undergoing a gradient alcohol dehydration, xylene clearing, and neutral gel fixation.

### Evaluation of staining

Five high-power fields were selected using a 200× lens, where the number of positive cells was counted and their percentages determined. The cells received scores based on the percentage of positive cells as follows: <5% (0 points), 5-25% (1 point), 26-50% (2 points), 51-75% (3 points), and >75% (4 points). Additionally, points were allocated according to staining intensity: strongly positive (3 points), moderately positive (2 points), and weakly negative (1 point). A score of >2 points was deemed positive, while ≤2 points was considered negative.

### Cell culture

A549 cells were cultured in Dulbecco's Modified Eagle's Medium (DMEM, HyClone, Logan, UT) supplemented with 10% fetal bovine serum (FBS, ExCell, Shanghai, China). The A549 cell line was incubated in a humidified chamber at 37 °C with 5% CO2.

### Silencing FOXA1 and HER2 by using small interfering RNA (siRNA)

FOXA1 and HER2 siRNAs, alongside their corresponding negative control (NC) siRNAs, were acquired from Shanghai GenePharma Co., Ltd. (Shanghai, China). Details of four FOXA1 and three HER2 siRNA sequences are provided in Table S1. Transfection of siRNAs was conducted using Lipofectamine™ 3000 Reagent (Thermo Fisher). Cells were harvested 72 hours post-transfection to assess the knockdown efficiency of FOXA1 and HER2 in A549 cells (n = 6) through western blotting and qPCR analysis. The siRNA demonstrating the highest efficiency was employed in further experiments.

### RNA isolation, reverse transcription, and qRT-PCR

RNA was extracted from the NSCLC cell lines, tissues, and adjacent non-cancerous tissues using Trizol (Takara Bio, Inc., Shiga, Japan). The ARF5 gene served as the internal control. Cycling conditions included an initial activation of DNA polymerase at 95 °C for 10 minutes, followed by 45 cycles of 95 °C for 15 seconds, 60 °C for 15 seconds (for FOXA1 and HER2), and 72 °C for 10 seconds. The specificity of the amplification products was validated through melting curve analysis. Experiments were conducted independently in triplicate. Specific sense primers for FOXA1, HER2, and ARF5 are provided in Supplementary Table 3.

### Colony formation assay

Cells were digested with 0.25% trypsin and subsequently seeded into a DMEM medium supplemented with 10% FBS at a density of 8×10² cells/well. Following two weeks of culture, visible colonies were fixed with paraformaldehyde for 20 minutes and stained with 0.1% crystal violet (JissKang, China). The number of colonies per group was then counted. Colony forming efficiency (%) = (number of colonies formed / number of cells seeded) × 100%.

### CCK-8

The cell viability was detected using a CCK-8 assay kit (Dojindo Molecular Technologies, Japan). Cells were seeded in 96-well plates at a density of 2×10³ cells/well, and 10 µL of CCK-8 solution was added after culturing for 0, 24, 48, and 72 hours. Following a 2-hour incubation at 37 ℃, absorbance at 450 nm was measured.

### MTT cytotoxicity assay

RC48 purchased from Rongchang Pharmaceuticals Co., Ltd., China. Drug sensitivity test was determined by MTT assay. Cells were seeded in 96-well plates in 100 μl RPMI-1640 medium supplemented with 10% FBS at 5×10^3^ cells/well. Once cells attached, they were treated with 12.5, 25, 50 or 100 µg RC48 (10mg/ml) and incubated at 37˚C in 5% CO2 for 48 hrs. Subsequently, 10 µl of MTT (5 mg/ml) (Sigma, St. Louis, MO, USA) was added to each well, and the plates were incubated at 37˚C for 4 hrs. At the end of incubation, supernatants were removed and 100 ul of DMSO (Sigma) was added to each well. The absorbance value (OD) of each well was measured at 490nm. The calculated rates were then used for curve fitting and half maximal inhibitory concentration (IC50) calculations. Experiments were carried out three times.

### Flow cytometry

1 × 10^6^ A549 cells were washed twice with PBS. Subsequently, 200 μL of binding buffer, 10 μL of FITC-labeled Annexin-V (20 μg/mL), and 5 μL of PI (50 μg/mL) were added. The cells were incubated in the dark at room temperature for 30 minutes. Following this, 400 μL of PBS was added, and the cells were analyzed using a FACScalibur cytometer and CellQuest Version 3.2.1 software. A tube lacking both Annexin V-FITC and PI served as a negative control. All experiments were conducted in triplicate.

### Cell cycle analysis

Cell cycle examination was carried out according to a previous description^28^. The DNA content of labeled cells was acquired using a FACS cytometry assay (BD Biosciences).

### Western blot analysis

Cells were lysed using RIPA buffer (Kaiji, Nanjing, China), and protein concentrations were determined via BCA assay (Beyotime Institute of Biotechnology, Haimen, China). A total of 30 µg of protein was separated on a 10% SDS-polyacrylamide gel electrophoresis (PAGE) gel and then electro-transferred onto polyvinylidene fluoride membranes (Invitrogen; Thermo Fisher Scientific, Inc., Waltham, MA, USA). Membranes were blocked using 5% non-fat dry milk (with bovine serum albumin for phosphorylation antibodies) in Tris-buffered saline (pH 7.5) containing 0.1% Tween-20, followed by overnight immunoblotting at 4 °C with primary antibodies (refer to Supplementary Table 4). The secondary antibodies, HRP-conjugated anti-rabbit (cat no. SA00001-2, 1:1,000) or anti-mouse IgG (cat no. SA00002-1, 1:1,000) from Protein Tech Group Inc., (Chicago, IL, USA), were applied for 1 hour at room temperature. Signals were detected using Enhanced chemiluminescence reagent (Pierce; Thermo Fisher Scientific, Inc.). Bands were analyzed using Image J, with protein expression quantified by the calculation: Integrated optical density (IOD) = density (mean) x area.

### Luciferase reporter assay

A Luciferase reporter assay was conducted to demonstrate that FOXA1 directly regulates HER2. The wild-type (WT) 3' UTR and mutant 3' UTR were cloned into psiCHECK-2 vectors. Both the WT and mutant 3' UTR vectors were co-transfected with either the FOXA1 plasmid/inhibitors or a nonspecific control into NSCLC cells. To further explore whether FOXA1 affects the promoter activity of HER2, we constructed the HER2 promoter-luciferase reporter plasmid, pGL3-HER2. Luciferase activity was measured 48 hours post-transfection using the Dual-Luciferase Reporter Assay System (Promega, Madison, WI, USA).

### CHIP assay

According to the previous description^28^, we utilized a ChIP assay kit (Millipore) to conduct ChIP assays following the manufacturer's instructions. DNA-protein complexes were immunoprecipitated from A549 cells using anti-HER2 antibodies or IgG (Cell Signaling Technology, Danvers, MA, USA). The HER2 promoter region was analyzed by qRT-PCR and PCR. PCR primers are listed in Table S3.

### Statistical analysis

Patient clinical data and follow-up information were systematically gathered. Statistical analyses were conducted using SPSS version 22.0. Measurement data were presented as means ± standard deviations, while categorical data were expressed as percentages. Rates were compared using the chi-square test. Survival curves were plotted using the Kaplan-Meier method, and differences in overall survival (OS) across groups were assessed using the log-rank test. Univariate and multivariate survival analyses were performed using the Cox proportional hazards model. Spearman's correlation coefficient was employed to examine the relationship between FOXA1 and HER2. A p-value of less than 0.05 was deemed statistically significant.

## Results

### Expression of FOXA1 gene in NSCLC tissues and PT tissues

**Figure 1:**
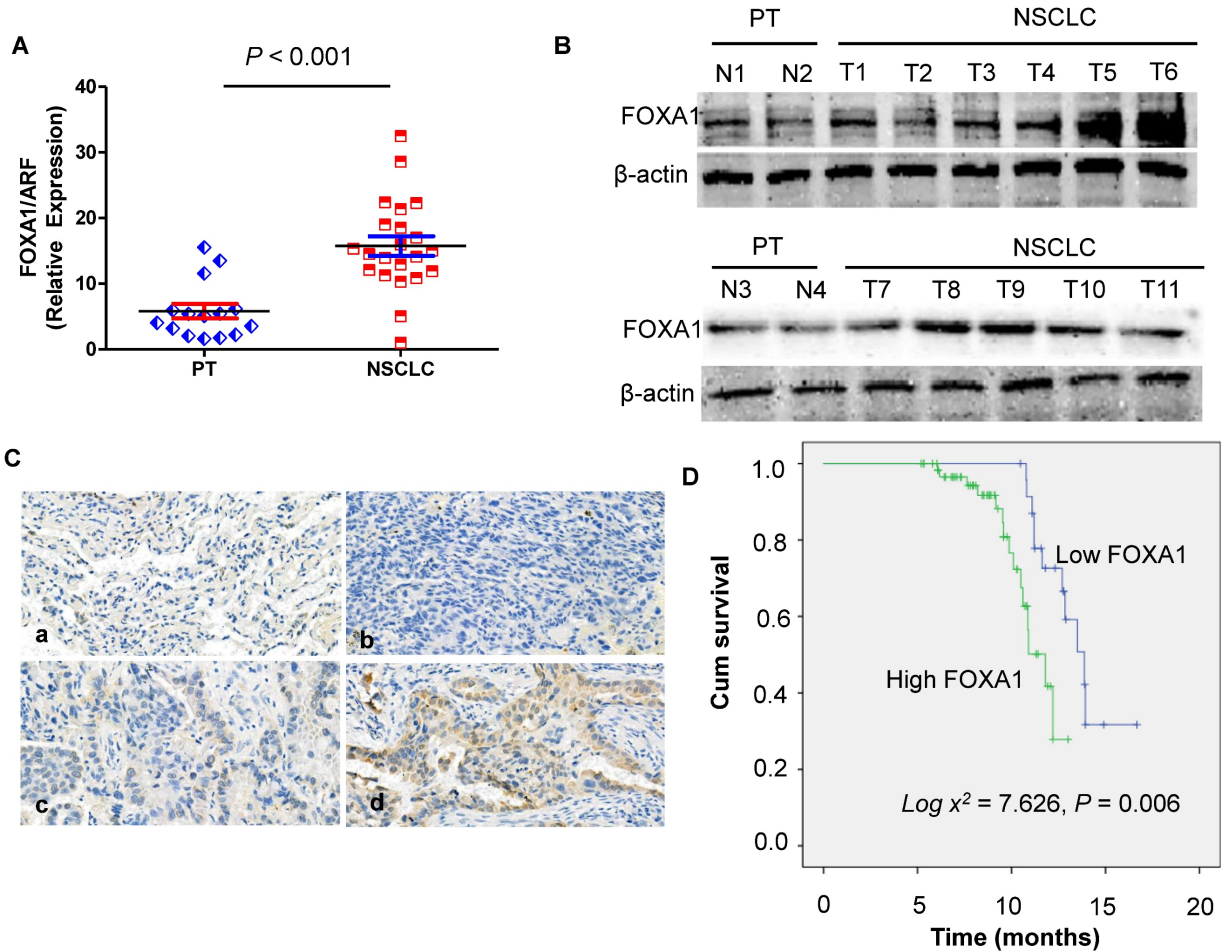
** Expression of FOXA1 gene in para-cancerous tissues (PT) and NSCLC. (A)** mRNA expression of FOXA1 is decreased in PT compared with NSCLC tissues by real-time PCR assay. Data are presented were presented as mean ± SD for three independent experiments (***P<0.001). **(B)** Western blot analysis of FOXA1 expression in 11 cases of NSCLC tissue samples compared with 4 PT tissues. **(C)** FOXA1 protein was expressed in NSCLC and PT samples (original magnification: 400X). A. a). Negative staining of FOXA1 in PT. b). Negative staining of FOXA1 in NSCLC. c) Weak staining of FOXA1 in NSCLC. d). Strong cytoplasmic FOXA1 staining in NSCLC. **(D)** Expression of FOXA1 protein predicts overall survival of NSCLC patients. Patients with positive FOXA1 had worse survival than those with negative FOXA1 (*P* = 0.006).

In order to assess the role of FOXA1 in NSCLC, we performed real-time PCR to measure the expression of FOXA1 mRNA transcripts in 22 freshly collected NSCLC tissues and 15 freshly collected para-cancerous tissues (PT) were analyzed. Compared with PT, NSCLC tissues demonstrated significantly elevated levels of FOXA1 mRNA (P < 0.001) (Figure 1A). FOXA1 protein expression was observed to be up-regulated in 11 cases of NSCLC relative to 4 PT tissues as determined by Western blot analysis (Figure 1B). These findings suggest that FOXA1 might function as an oncogene in NSCLC. To further elucidate the role of FOXA1 in NSCLC, we assessed its expression levels and subcellular localization in 87 archived paraffin-embedded NSCLC samples via immunohistochemical staining (Figure 1C). The average follow-up period for all participants was 14.23 months (range, 6.5 to 17.8 months). By the conclusion of the study, 43 patients (49.43%) had succumbed to disease progression of malignant tumors, while 44 remained alive. Patient demographic and clinical characteristics for the cohort are summarized in Table S1.

### FOXA1 high expression is associated with overall survival of NSCLC

To examine the prognostic significance of FOXA1 expression in NSCLC, we evaluated the correlation between tumor FOXA1 expression levels and patient survival using Kaplan-Meier analysis and the log-rank test. Among 87 NSCLC cases with available prognostic data, we found that FOXA1 protein expression levels were significantly associated with overall survival. Patients with high FOXA1 expression exhibited poorer prognoses compared to those with low expression (Figure 1D) (*P* = 0.006).

### Reduced FOXA1 expression inhibits NSCLC cell proliferation and promotes apoptosis

**Figure 2:**
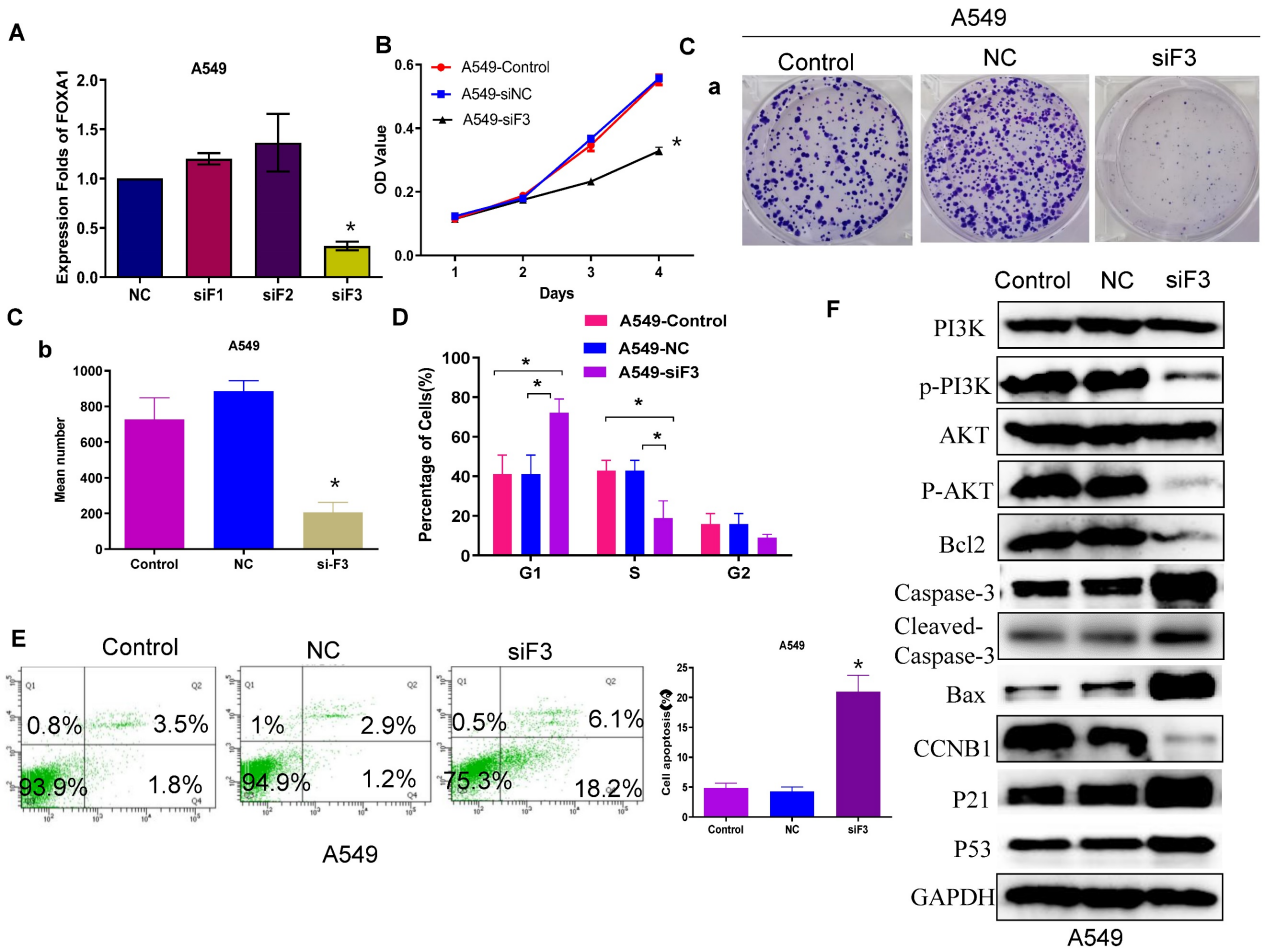
** FOXA1 promotes tumor cell proliferation and apoptosis. (A)** FOXA1 expression levels in A549 cell with knockdown of FOXA1 detected by qRT-PCR. **(B)** CCK-8 assay analyzed the effect of silencing FOXA1 on cell viability. **(C)** Cell cycle of A549 cell transfected with FOXA1 inhibitors (n = 3). **(D)** Colony formation assay analyzed the effect of silencing FOXA1 on cell proliferation. **(E)** A549 cell apoptosis after si-FOXA1 treatment detected by fow cytometry. **(F)** Enrichment of cell cycle-related and apoptosis-associated proteins in the cotransfected cells was determined using western blotting. Student's t-test. mean± SD (*P <0.05).

Previous research has confirmed that FOXA1 is highly expressed in lung cancer^20^-^25^, and our earlier findings align with this observation. In the current study, we further investigated the role of FOXA1 in lung cancer. Quantitative RT-PCR demonstrated that si-FOXA1 significantly diminished FOXA1 mRNA expression in cells relative to controls (si-NC) (Figure 2A). We conducted a CCK8 assay (Figure 2B) and a colony formation assay (Figure 2C) to examine the impact of FOXA1 on NSCLC cell proliferation. These assays indicated that reduction of FOXA1 inhibits cell proliferation *in vitro*. Flow cytometry analysis was employed to assess the influence of FOXA1 on the cell cycle of NSCLC cells. Decreased expression of FOXA1 led to a 20% increase in G1 phase cell numbers in the A549 cell line (*P* < 0.05), with corresponding reductions in both S phase and G2 phase percentages compared to controls (Figure 2D). These findings suggest that FOXA1 inhibition induces cell cycle arrest by blocking cells in the G1 phase, thereby reducing cell numbers in the S and G2 phases.

The level of apoptosis following si-FOXA1 treatment was assessed using flow cytometry. FOXA1 knockdown increased the apoptosis rate in A549 cells compared to those transfected with si-NC and the control cells (Figure 2E). Western blot analysis showed higher levels of P21 and P53 in A549 cells with reduced FOXA1 expression, in contrast to control and si-NC cells, whereas CCNB1 levels were decreased. Moreover, FOXA1 knockdown diminished Bcl2 expression while elevating levels of Bax, caspase 3 and cleaved caspase 3 in A549 cells. Additionally, the expression levels of P-AKT and P-PI3K were significantly reduced following FOXA1 interference, although the expression levels of AKT and PI3K remained unchanged (Figure 2F). These results suggest that FOXA1 may facilitate the proliferation of lung cancer and inhibit apoptosis by modulating the PI3K/AKT signaling pathway and its downstream effectors involved in the cell cycle and apoptosis.

### FOXA1 binds the promoter region of HER2

**Figure 3:**
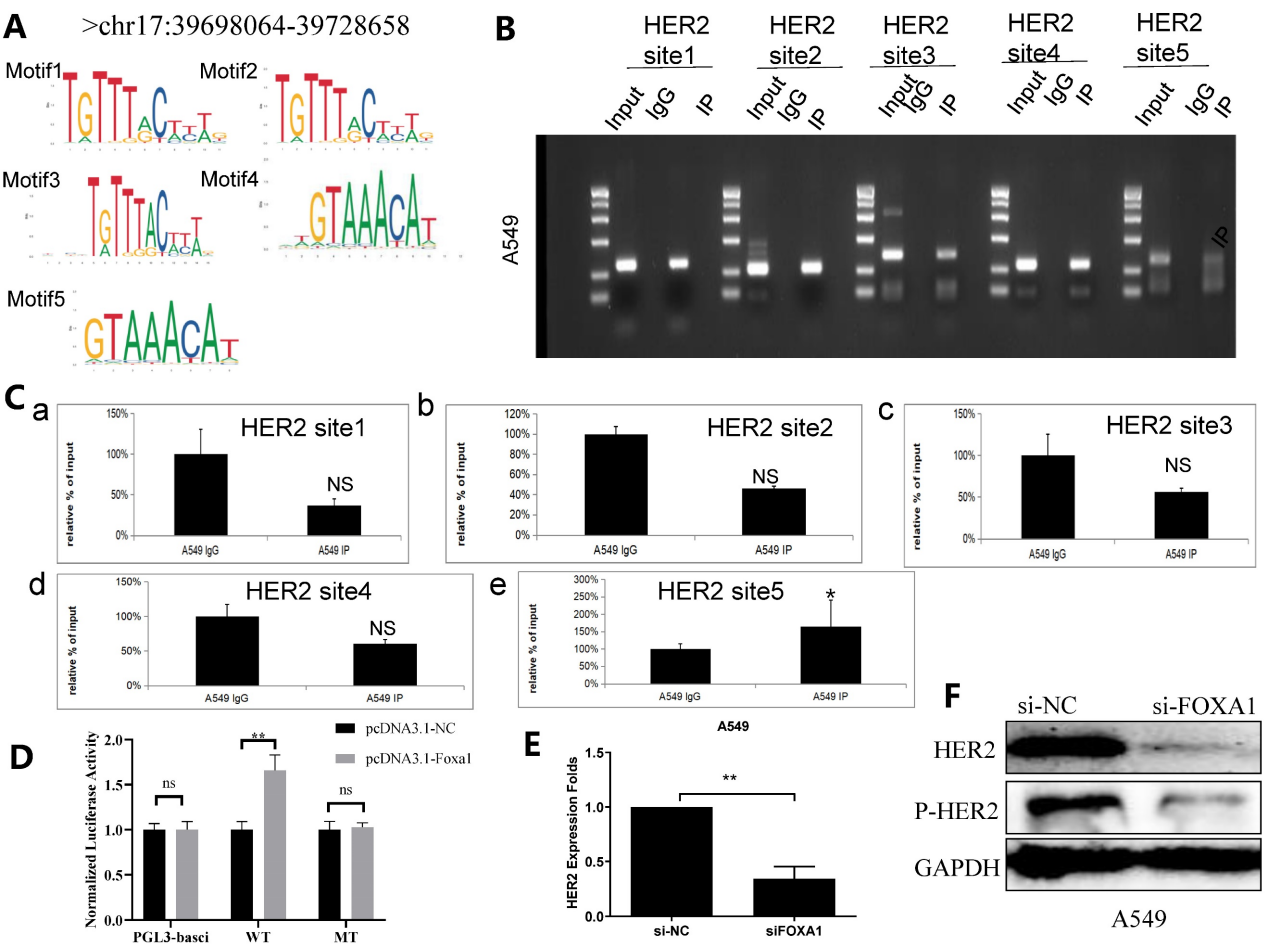
** FOXA1 binds to a specific sequence within the HER2 gene promoter. (A)** The ENCODE project (https://www.encodeproject.org/,accessed on 1 March 2024), TRANSFAC and the TFFFIND search tool from the Piptools package were used to identify segments of the HER2 proximal gene promoter in NSCLC cells that contained binding sequences for FOXA1. Five such sequences were identified (sequence logo for the FOXA1 consensus motif shown, accessed on 1 March 2024), of which one was synthesized as oligonucleotides for binding assays. **(B)** CHIP experiment shows 5 binding sites of FOXA1 in HER2 promoter region. **(C)** Amplification of HER2-binding sites after Ch-IP using an antibody against FOXA1. An IgG antibody was used as the negative control. **(D)** Relative luciferase activity of the indicated promoter vectors A549 cell transfected with FOXA1 plasmids. **(E)** HER2 mRNA expression with the treatment of silencing FOXA1 as analyzed by qRT-PCR. **(F)** HER2 protein expression with the treatment of silencing FOXA1 as analyzed by western blot; Student's t-test. mean± SD (*P <0.05, *** *P* <0.001).

A subsequent targeted bioinformatics analysis was conducted to identify potential sequences where FOXA1 binds to the HER2 gene promoter. Utilizing data from the ENCODE project (https://www.encodeproject.org/, accessed on 1 March 2024), extensive regions of FOXA1 binding on the HER2 promoter were delineated through analysis of CHIP-seq information derived from A549 cells. As those regions are extensive and of impractical use for *in vitro* binding assays, the search for specific FOXA1 binding sites within the HER2 promoter was refined using transcription factor binding sites (TFBS) data from TRANSFAC and the PipTools package^26^,^27^. This narrowed analysis produced a list of five candidate sequences in regions presumed to involve FOXA1 binding to the HER2 promoter (Figure 3A; highlighted sequences). Chromatin immuno-precipitation (ChIP) analysis confirmed that FOXA1 was most significantly bound to TFBS 5 of the HER2 promoter (Figure 3B and C), indicating a direct interaction. Further validation was carried out using the dual luciferase assay, which demonstrated that deletion of FOXA1 substantially reduced the luciferase activity associated with HER2-WT (Figure 3D). Additionally, qRT-PCR and western blot assays indicated that silencing FOXA1 significantly decreased HER2 expression, and also led to considerable downregulation of p-HER2 protein levels (Figure 3E, 3F). These findings collectively suggest that FOXA1 transcriptionally activates HER2 expression.

### The anti-tumor mechanisms of RC48 were assessed in terms of cell cycle arrest, apoptosis induction, and cellular signaling interference

**Figure 4:**
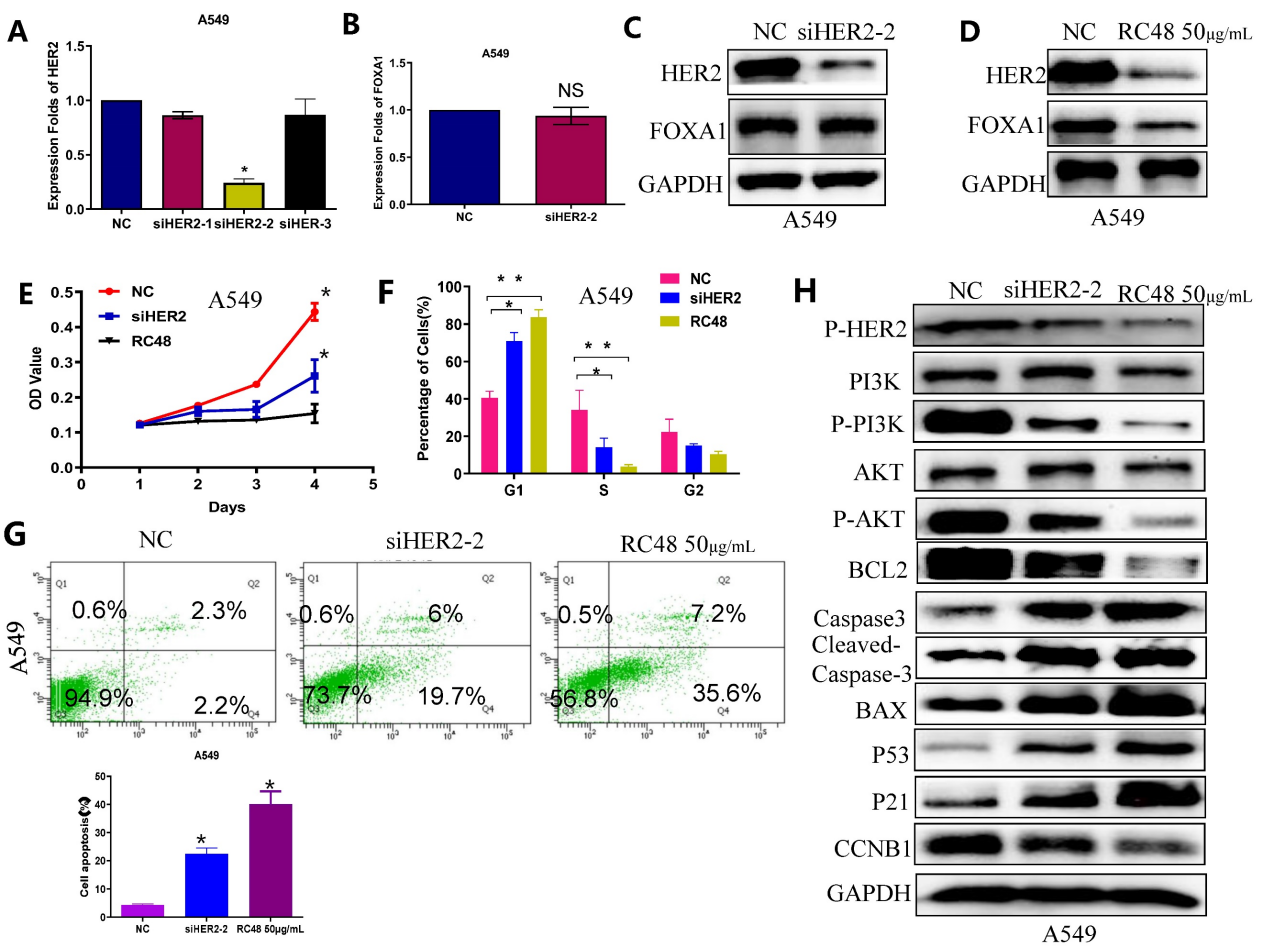
** Effects of RC48 on cell cycle arrest, apoptosis induction and suppression of HER2-mediated cell signaling. (A)** HER2 expression levels in A549 cell with knockdown of HER2 detected by qRT-PCR. **(B)** FOXA1 expression levels in A549 cell with knockdown of HER2 detected by qRT-PCR. **(C)** HER2 and FOXA1 protein expressions with the treatment of silencing HER2 as analyzed by western blot. **(D)**HER2 and FOXA1 protein expressions with the treatment of RC48 as analyzed by western blot. **(E)** CCK-8 assay analyzed the effect of RC48 and knockdown of HER2 on cell viability. **(F)** A549 cell cycle arrest results analyzed by FCM. A549 cells were treated with siHER2 and RC48. **(G)** A549 cell apoptosis after si-HER2 and RC48 treatment detected by fow cytometry. **(H)** Representative Western blots results of CCNB1, P21, P53, Caspase3, Cleaved-Caspase3, Bcl2 and Bax in the A549 cells. Interference of HER2 signal pathway related proteins, P-HER2 and P-AKT, detected by Western blot. Student's t-test, mean ± SD (*P <0.05, ** *P* <0.001).

HER2, a transmembrane glycoprotein receptor, is implicated in cellular processes including growth, differentiation, migration, and survival. The primary observed alterations in non-small cell lung cancer (NSCLC) related to HER2 consist of mutations, amplifications, and overexpression, with exon 20 insertion mutations being the most prevalent^29^. Moreover, HER2 mutations and amplifications contribute to acquired resistance in NSCLC patients treated with EGFR-TKIs. Historically, chemotherapy has been the principal treatment for NSCLC patients harboring HER2 mutations; however, its effectiveness has been disappointing. Previous research indicates that FOXA1 can bind to the promoter region of HER2 and enhance its expression. Our findings further reveal that interference with HER2 does not alter FOXA1 expression at the mRNA or protein levels (Figures 4B and 4C), confirming that HER2 interference does not mediate its antitumor effect through the regulation of FOXA1. Trastuzumab deruxtecan (T-DXd), a novel antibody-drug conjugate (ADC) targeting HER2, has received FDA approval for treating HER2-mutated, unresectable, or metastatic NSCLC in adults following systemic therapy. The mechanism of another ADC, RC48, in lung cancer remains understudied. Our results demonstrate that RC48 not only reduces HER2 protein levels in lung cancer cells but also decreases FOXA1 protein expression (Figure 4D). CCK8 assays indicate that both HER2 reduction and RC48 treatment inhibit cell proliferation *in vitro*, with RC48 showing a more significant effect (Figure 4E). Flow cytometry analysis reveals that siHER2 and RC48 impede the G1 to S cell cycle transition in A549 cells (Figure 4F) and increase apoptosis rates, with RC48 causing a greater increase in apoptosis compared to the negative control (Figure 4G).

Interference with HER2 signaling pathway-related proteins was detected by immunoblotting (Figure 4H). The results showed that both RC48 and siHER2 significantly decreased the levels of p-HER2, p-AKT, and p-PI3K compared to the NC. Additionally, the expression of cell cycle and apoptosis-related factors downstream of the PI3K/AKT signaling pathway was detected. Compared to the control group, the siHER2 group and the RC48 group exhibited a significant increase in the expression of P21, P53, and BCL2, and a decrease in CCNB1, caspase3, and Cleaved caspase 3 and Bax expression. The regulatory effect of RC48 appeared to be more substantial. These data suggest that the antitumor activity of RC48 is HER2-directed and results from the inhibition of intracellular signaling via the PI3K/AKT pathway (Figure 4H).

### Relationship between FOXA1 and HER2 expression and clinicopathological features

**Figure 5:**
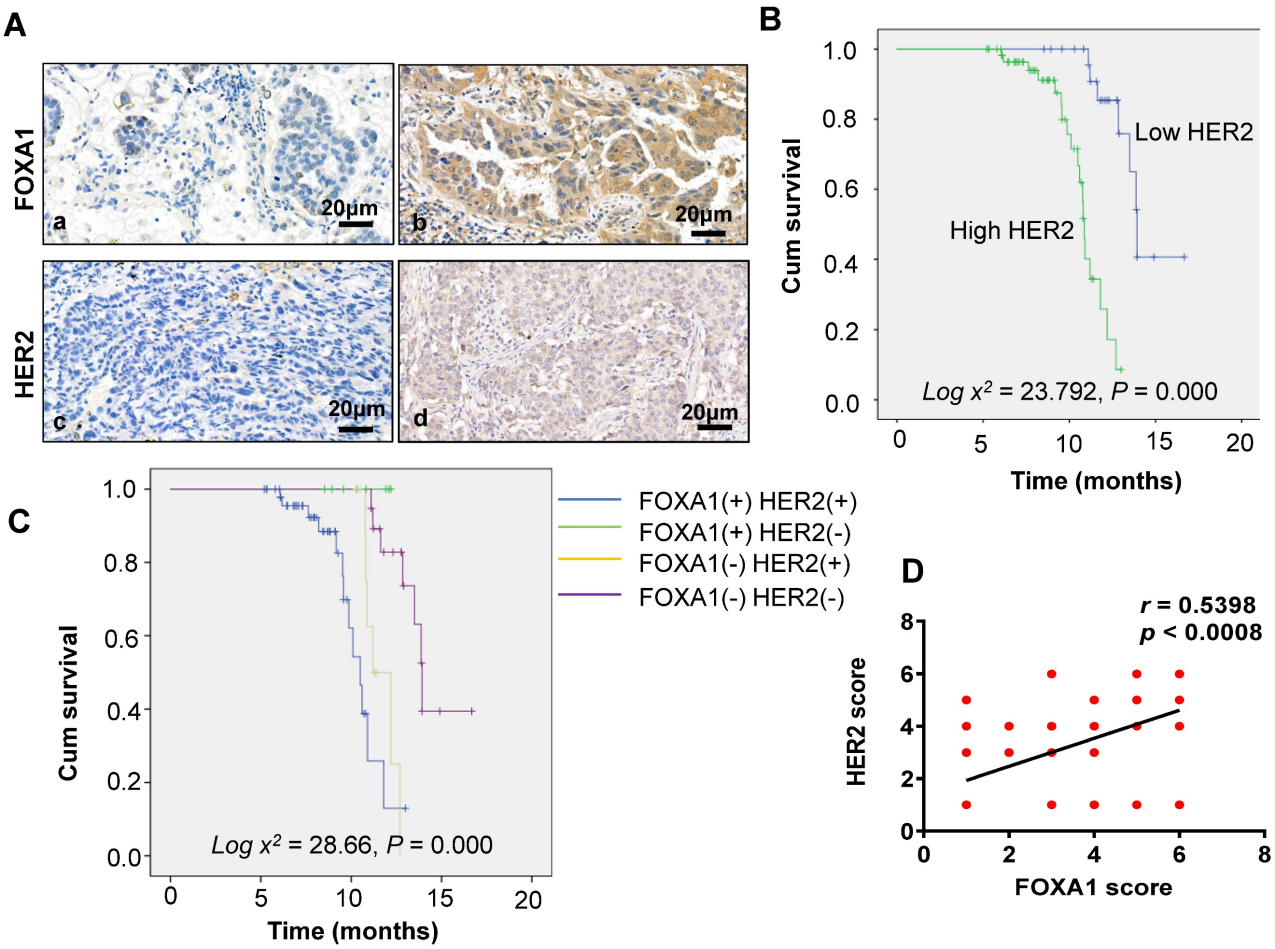
**(A)** Immunohistochemical staining of HER2 and FOXA1 in adenocarcinoma (×40): a: FOXA1 negative b: FOXA1 positive c:HER2 negative d: HER2 positive. **(B)** Overall survival in groups with HER2 expression. **(C)** Overall survival in different groups. **(D)** Correlations between FOXA1and HER2 expression levels were calculated. Two tailed Spearman's correlation analysis ( r= 0.5398, *P* < 0.0001).

Of the 87 patients with NSCLC, 58 (66.67%) tested positive for FOXA1, and 60 (68.97%) tested positive for HER2, as depicted in Figure 5A. FOXA1 and HER2 can be expressed not only in the nucleus but also in the cytoplasm. According to Table 1, the positive expression of FOXA1 and HER2 correlates significantly with tumor differentiation and distant metastasis (P = 0.032 and *P* < 0.001, respectively). However, there was no significant association with age, sex, pathological type, or smoking history (*P* > 0.05 for all).

### Correlation analysis between FOXA1 and HER2 expression and patient prognosis

The relationship between FOXA1 and HER2 expression and overall survival (OS) in the general population was examined using the Kaplan-Meier method (Figure 5B). Survival durations were 11.16 months for patients with positive FOXA1 expression and 13.84 months for those with negative expression (Figure 1D). Similarly, for HER2, the survival times were 10.82 months with positive expression and 14.51 months with negative expression. Patients with negative expressions of both FOXA1 and HER2 exhibited superior prognoses compared to those with positive expressions (log χ2 = 7.626, P = 0.006 and log χ2 = 23.792, P = 0.000, respectively). Additionally, patients were categorized into four groups based on FOXA1 and HER2 expression: the FOXA1 (-) and HER2 (-) group (double-negative), the FOXA1 (+) only group, the HER2 (+) only group, and the FOXA1 (+) and HER2 (+) group (double-positive) (Figure 5C). The best prognosis was observed in the double-negative group, whereas the other three groups experienced poorer outcomes (log χ2 = 28.600, *P* = 0.000). Correlation analysis revealed a positive association between FOXA1 and HER2 expression in lung cancer (r = 0.5398, *P* < 0.0001), confirming the findings at the histological level (Figure 5D).

### Univariate and multivariate analyses of patient prognosis

The effects of clinicopathological parameters on prognosis were analyzed using the Cox proportional hazards model (Table 2). Univariate analysis indicated that positive FOXA1 and HER2 expression, distant metastasis, and differentiation were risk factors affecting patient prognosis. Multivariate analysis further revealed that positive FOXA1 and HER2 expression, along with distant metastasis, were independent risk factors influencing patient prognosis (all, *P* < 0.05).

## Discussion

FOXA1, a member of the FoxA family, is frequently studied in tumor research due to its mediation of histone H1 and DNA genome demethylation, resulting in chromatin relaxation in densely stained regions^30^. It also enhances transcriptional activities and is critical as a pioneer factor in cell differentiation and development^31^. Recent findings indicate a strong correlation between FOXA1 and tumor resistance. Enhanced FOXA1 expression has been linked to decreased sensitivity of estrogen receptor-positive breast cancer to chemotherapy, serving as a predictor of poor prognosis in patients undergoing neoadjuvant chemotherapy for this cancer type^32^. Consequently, increased FOXA1 expression may correlate with the adverse outcomes of malignant tumors. Wang's systematic analysis of extensive ChIP-Seq transcription factor data from A549 cell lines revealed that FOXA1 plays a pivotal role in the early stages of lung cancer metastasis by influencing epithelial-mesenchymal transition (EMT)^23^. Zhang identified FOXA1 as an oncogene in lung squamous carcinoma cells^25^. This study further analyzed the expression and prognostic significance of FOXA1 in lung cancer patient tissues, discovering that patients with positive FOXA1 expression exhibited poorer prognoses compared to those negative for FOXA1. Remarkably, 66.67% of lung cancer tissue samples showed positive FOXA1 expression, indicating a substantial increase in its levels. Real-time PCR assays and Western blot analysis confirmed that FOXA1 expression is reduced in post-treatment (PT) samples compared to non-small cell lung cancer (NSCLC) tissues. The balance between pro-apoptotic proteins and anti-apoptotic proteins within the cell mediates apoptosis, among which Bcl-2 and Bax are the more extensively studied pro-apoptotic and anti-apoptotic proteins. They are usually present in normal cells as dimers, which can effectively regulate cell apoptosis. The ratio of these two proteins mediates the progress of the cell into the apoptosis phase. Literature shows that increased expression of Bcl-2 can bind with Bax to form heterodimers, inhibiting Bax activity and preventing apoptosis; increased Bax expression can bind with Bax to form homodimers, activating caspase-9 to cleave pro-caspase-3, and the activation of caspase-3 expression facilitates this cleavage, thereby triggering a caspase cascade reaction, ultimately inducing apoptosis 33-35. The PI3K/Akt pathway is an intracellular signaling pathway that promotes metabolism, proliferation, cell survival, growth, and angiogenesis in response to extracellular signals 36. Akt enhances cell survival by inhibiting the function of pro-apoptotic proteins and the apoptotic process. Akt negatively regulates the function or expression of Bcl-2 family members, Bax protein, and Bim protein. P53 is also a cancer gene that mediates apoptosis. Akt can promote the degradation of p53 by phosphorylating MDM2 37. Akt can also phosphorylate P21/Waf1/Cip1 and P27/Kip2, and inhibits their anti-proliferative effect by retaining them in the cytoplasm 38. Thus, it can promote cells to enter the cell cycle for proliferation. Functional and mechanistic investigations have confirmed that FOXA1 promotes lung cancer cell proliferation and inhibits apoptosis by modulating the PI3K/AKT signaling pathway and its downstream effectors involved in the cell cycle and apoptosis. Thus, it is speculated that FOXA1 may function as an oncogene in lung cancer.

In non-small cell lung cancer (NSCLC), HER2 serves as an oncogenic driver gene. HER2 mutations, which occur in 2% of lung cancer cases, enhance EGFR signaling, thereby promoting differentiation, proliferation, and metastasis of tumor cells^5^. Previous studies have identified FOXA1 as a mediator of transcriptional responses to HER2-targeted therapy in breast cancer, with the ability of FOXA1 protein to bind to the HER2 gene promoter and influence its transcription^26^, ^27^. This study also demonstrates that FOXA1 binds to the promoter region of HER2 and promotes its transcription in lung cancer. Interference with FOXA1 results in a marked decrease in HER2 expression at both mRNA and protein levels. Conversely, interfering with HER2 does not significantly affect the expression of FOXA1 at the mRNA and protein levels, confirming that FOXA1 can regulate the expression of HER2, but not vice versa.

In addition to targeted therapies, ADCs demonstrate potent anti-tumor activity by targeting the HER family^39^. Unlike monoclonal antibodies and tyrosine kinase inhibitor (TKI) drugs, ADC drugs exhibit a lower reliance on HER2 and its downstream signaling pathways for tumor cell eradication^40^. ADC drugs not only inhibit the relevant signaling pathways by binding to the HER2 protein but also depend significantly on the cytotoxic payload released upon internalization into tumor cells for cell destruction. Therefore, ADC drugs may surmount resistance to HER2-targeted therapy caused by traditional pathway activation. Disitamab vedotin (RC48) is an innovative ADC that links Hertuzumab (a novel anti-HER2 monoclonal antibody) with monomethyl auristatin E (MMAE) via cleavable linkers^41^. RC48 has been approved for the treatment of HER2-positive cancer patients who have undergone at least two prior systemic chemotherapy regimens for locally advanced or metastatic gastric cancer (GC), gastroesophageal junction cancer (GEJC), and urothelial carcinoma (UC)^12^-^15^. RC48 precisely recognizes and binds to tumor cells, subsequently inducing cell death by penetrating the cell membrane. Interestingly, while HER2 does not regulate the expression of FOXA1, the introduction of RC48 significantly reduces the expressions of both HER2 and FOXA1 in lung cancer cells, indicating that RC48 can modulate both HER2 and FOXA1 expressions. Functional studies further reveal that both RC48 and siHER2 can inhibit lung cancer cell proliferation and promote apoptosis, with RC48 showing a slightly stronger effect. Further mechanistic research confirms that RC48 not only regulates the HER2/PI3K/AKT signaling pathway but also the FOXA1/HER2/PI3K/AKT signaling pathway, exerting a potent anti-tumor effect. Our research confirms that compared to merely inhibiting HER2, the ADC drug RC48 possesses robust and persistent anti-tumor activity, presenting a novel therapeutic option for lung cancer patients.

The factors impacting patient prognosis were analyzed utilizing the Cox proportional hazards model. The results of the univariate analysis indicated that positive expression of FOXA1, HER2 expression, and distant metastasis of FOXA1 are risk factors affecting prognosis. Conversely, the multivariate analysis demonstrated that only the positive expression of FOXA1 and HER2, alongside distant metastasis, were independent risk factors for prognosis. Distant metastasis is considered a determinant of poor prognosis in patients with advanced lung cancer in clinical settings, aligning with the findings of this study. Additionally, a positive correlation between FOXA1 expression and HER2 expression was confirmed.

In summary, a correlation exists between FOXA1 and HER2 in the tumor tissues of patients with advanced NSCLC. Elevated expression levels of both FOXA1 and HER2 are associated with a poor prognosis. FOXA1 can translocate to the nucleus and initiate the transcription of HER2, potentially leading to increased HER2 protein expression and subsequent tumorigenic signaling through pathways that include PI3K and AKT activation. While HER2 does not regulate FOXA1 expression, RC48 can influence the HER2/PI3K/AKT signaling pathway by modulating FOXA1 expression. Beyond traditional HER2 or FOXA1-targeted therapies, ADC drugs such as RC48 could offer significant benefits to lung cancer patients and potentially transform the future treatment paradigm for lung cancer. Of course, we also need to pay attention to the toxicity of RC48.

## Supplementary Material

Supplementary tables.

## Figures and Tables

**Figure 6:**
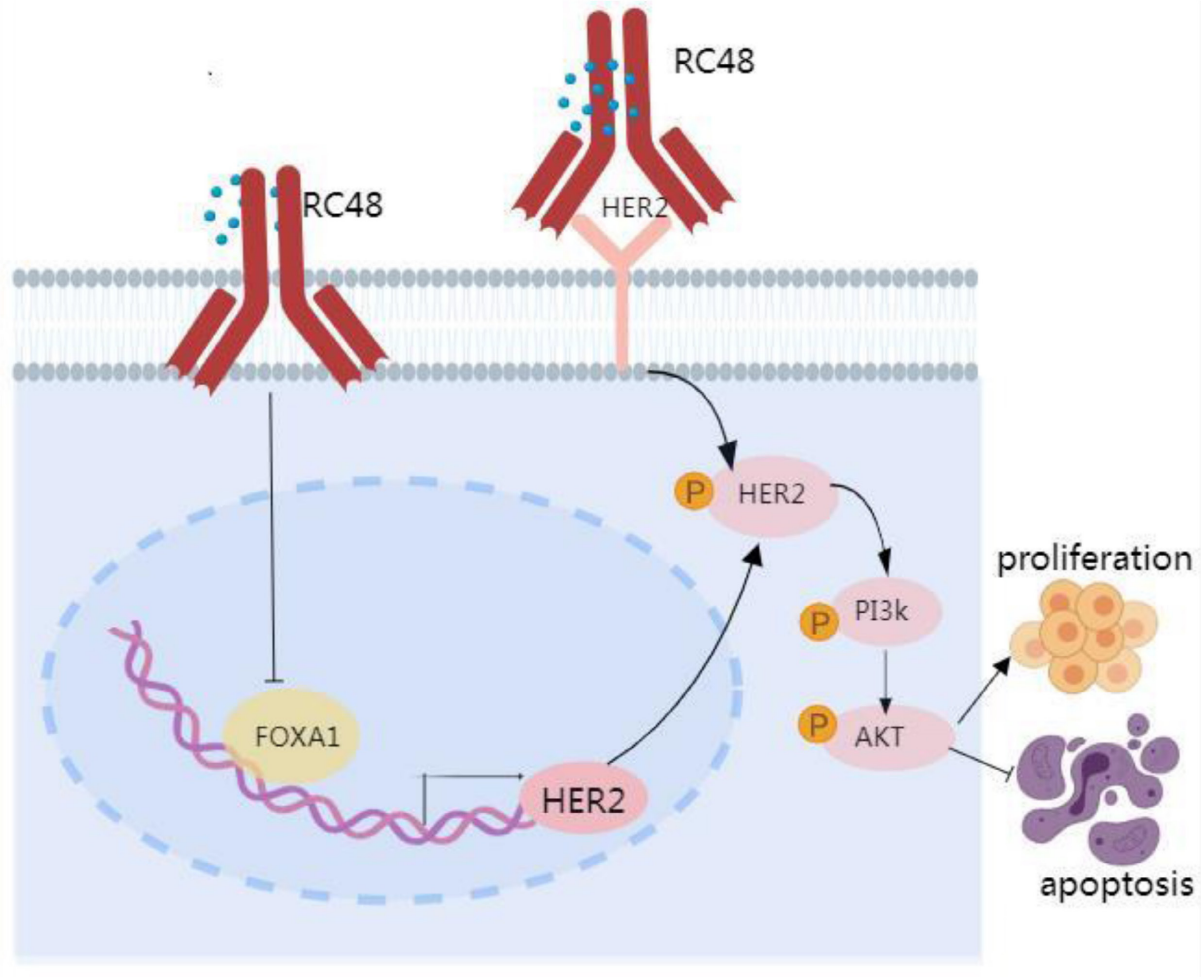
** Proposed model linking FOXA1 and HER2 in NSCLC.** FOXA1 is capable of translocating to the nucleus and turning on the transcription of HER2, whereupon a subsequent upregulation of HER2 protein expression would be predicted to drive tumorigenic signaling via pathways including the activation of PI3K and AKT. HER2 cannot regulate the expression of FOXA1, but the targeted drug RC48 can affect the HER2/PI3K/AKT signaling pathway by regulating the expression of FOXA1.

**Table 1:** Relationship between FOXA1 and HER2 expression and clinicopathological characteristics (n, %).

Characteristics	FOXA1 expression				HER2 expression			
	Positive	Negative	*χ2*	*P*	Positive	Negative	*χ2*	*P*
Sex								
Male	35 (62.50)	21 (37.50)	1.228	0.268	38 (63.33)	22 (36.67)	0.530	0.466
Female	23 (74.19)	8 (25.81)			22 (70.97)	9 (29.03)		
age								
≥65	25 (67.57)	12 (32.43)	0.024	0.878	29 (78.38)	8 (21.62)	2.655	0.103
<65	33 (66.00)	17 (34.00)			31 (62.00)	19 (38.00)		
Differentiation								
Well	7 (38.89)	11 (61.11)	7.888	0.019	8 (44.44)	10 (55.55)	6.909	0.032
Moderate	22 (73.33)	8 (26.67)			24 (80.00)	6 (20.00)		
Poorly	29 (74.36)	10 (25.64)			28 (71.79)	11 (28.21)		
M								
No	14 (43.75)	18 (56.25)	11.960	0.000	15 (46.88)	17 (53.12)	11.540	0.000
Yes	44 (80.00)	11 (20.00)			45 (81.82)	10 (18.18)		
Pathological typing (n, %)								
Squamous cell carcinoma	45 (72.58)	17 (27.42)	3.763	0.153	44 (70.97)	18 (29.03)	0.618	0.734
Adenocarcinoma	10 (55.56)	8 (44.44)			12 (66.67)	6 (33.33)		
Adenosquamous carcinoma	3 (42.86)	4 (57.14)			4 (57.14)	3 (42.86)		
Smoking (n, %)								
Yes	25 (64.10)	14 (35.90)	0.209	0.647	26 (66.67)	13 (33.33)	0.175	0.676
No	33 (68.75)	15 (31.25)			34 (70.83)	14 (17.95)		

**Table 2:** Univariate and multivariate analysis of the influence of various clinicopathological characteristics on the prognosis of patients.

Characteristics	Univariate analysis			multivariate analysis		
	HR	95% CI	*P*	HR	95% CI	*P*
FOXA1positive	5.261	2.587~10.271	0.003	3.872	1.316~7.513	0.019
HER2positive	5.482	2.196~10.458	0.002	3.573	1.287~6.963	0.022
Age	1.021	0.344~1.652	0.263			
Sex	1.011	0.302~1.633	0.251			
Smoking	0.961	0.308~1.414	0.422			
Differentiation	2.416	1.018~4.784	0.035	1.022	0.353~1.823	0.054
Pathological typing	0.984	0.325~1.514	0.341			
M	4.754	2.301~8.423	0.021	2.781	1.443~4.918	0.031
