# Supplementary material for: FOXA1, induced by RC48, regulates HER2 transcription to enhance the tumorigenic capacity of lung cancer through PI3K/AKT pathway
Source: J Cancer. 2024 Sep 16;15(18):5863–75. doi: 10.7150/jca.100210 (PMC11493013; doi:10.7150/jca.100210)
Supplement: Supplementary file 1 — Supplementary tables. [file jcav15p5863s1.pdf]

Table S1 Basic characteristics of the patients

Characteristics	Value
Age	56.43±5.87
≥65	37（42.53）
<65	50（57.47）
Sex	
Male	56（64.37）
Female	31（35.63）
Differentiation（n,%）	
Well	18（20.69）
Moderate	30（34.48）
Poorly	39（44.83）
M（n,%）	
No	32（36.78）
Yes	55（63.22）
Pathological typing（n,%）	
Squamous cell carcinoma	62（71.26）
Adenocarcinoma	18（20.69）
Adenosquamous carcinoma	7（8.05）
Smoking（n,%）	
Yes	39（44.83）
No	48（55.17）

Table S2. The sequences used in this study.

Gene		Sequence	Company
siHER2-1	Sense	5'-CUGGCUCCGAUGUAUUUGATT-3'	GenePharma, Shanghai, China
	Antisense	5'-UCAAAUACAUCGGAGCCAGTT-3'	
siHER2-2	Sense	5' CCUUCGACAACCUCUAUUATT 3'	
	Antisense	5' UAAUAGAGGUUGUCGAAGGTT3'	
siHER2-3	Sense	5'GUGGAGUUAUGGUGUGACUTT3'	
	Antisense	5'AGUCACACCAUAACUCCACTT3'	
siHER2-4	Sense	5'GUCUUUGGGAUCCUCAUCATT3'	
	Antisense	5'UGAUGAGGAUCCCAAAGACTT3'	
Negative control	Sense	5'-UUC UCC GAA CGU GUC ACG UTT-3'	
	Antisense	5'-ACG UGA CAC GUU CGG AGA ATT-3'	
siFOXA1-1	Sense	5′-GCGACUGGAACAGCUACUATT-3′	
	Antisense	5′-UAGUAGCUGUUCCAGUCGCTT-3′	
siFOXA1-2	Sense	5′-CCACUCGCUGUCCUUCAAUTT-3′	
	Antisense	5′-AUUGAAGGACAGCGAGUGGTT-3′	
siFOXA1-3	Sense	5′-GCACUGCAAUACUCGCCUUTT-3′	
	Antisense	5′-AAGGCGAGUAUUGCAGUGCTT-3′	

Table S3.The primers used in this study.

Primers name		Sequence (5'-3')	Company
FOXA1	Forward	GCGCGAATTCAACCACCCGTTCTCCATCAA	Invitrogen, Shanghai, China
	Reverse	GCGCCTCGAGTCATTGGTAGTACGCCGGCTCC AG	
ARF5	Forward	ATCTGTTTCACAGTCTGGGACG	
	Reverse	CCTGCTTGTTGGCAAATACC	
HER2	Forward	ACG TTT GAG TCC ATG CCC AA	
	Reverse	AGG TAG TTG TAG GGA CAG GCA	
Promoter of HER2-A	Forward	ATGTGACTGTCTCCTCCCAAAT	keanxiang, Shanghai, China
	Reverse	GGATGTGTTGTGTTTACCTTGTG	
Promoter of HER2-B	Forward	GCATAGCAACCTGTCCCACA	
	Reverse	CCCATCTCCCACACCTCTTTAC	
Promoter of HER2-C	Forward	CATAGCAACCTGTCCCACAAG	
	Reverse	ACACCAGCATCTCTTTCTCTCT	
Promoter of HER2-D	Forward	AGCCACAAGGTAAACACAACACAT	
	Reverse	CCTACTCCATCCCAAGCCTATTTG	
Promoter of HER2-E	Forward	CCTGGAAGCCACAAGGTAAACA	
	Reverse	CATCCTACTCCATCCCAAGCCT	

Table S4. A list of antibodies used for WB, ChIP, and IHC.

Antibodies name	Cat. No	Company	Species	Dulution
FOXA1	53528	CST	Rabbit	1:400 (IHC); 1:1000 (WB); 1:100 (IF)
HER2	58613	CST	Rabbit	1:1000 (WB); 1:100 (IF)
Phospho-AKT	Ser473, 9271	CST	Rabbit	1:1000 (WB)
AKT	pan, 40D4, 2920	CST	Rabbit	1:1000 (WB)
ZEB1	3396	CST	Rabbit	1:1000 (WB)
PI3K	97885	CST	Rabbit	1:1000 (WB)
P-PI3K	p85 (Tyr458)/p55 (Tyr199)4228s	CST	Rabbit	1:1000 (WB)
P-HER2	Tyr1221/1222(2243)	CST	Rabbit	1:1000 (WB)
P21	ab109520	Abcam	Rabbit	1:1000 (WB)
P53	ab26	Abcam	Mouse	1:1000 (WB)
BCL2	ab32124	Abcam	Rabbit	1:2000 (WB)
Caspase3	ab32042	Abcam	Rabbit	1:500 (WB)
Cleaved -Caspase3	9669S	CST	Rabbit	1:500 (WB)
Bax	ab32503	Abcam	Rabbit	1:1000 (WB)
β-actin	60008-1-Ig	Proteintech	Mouse	1:5000 (WB)
IgG	2729	CST	Rabbit	1mg/ml(CHIP)
